# Alternative protocols for sanitizing hatching eggs and their effects on the microbiota of eggshell surface and chick yolk sac

**DOI:** 10.1007/s42770-026-01896-x

**Published:** 2026-03-06

**Authors:** Erica Faria Melo, Winnie Luiza Santos Clímaco, Mariana Andre Pompeu, Mariana Cristina Vieira, Henrique César Pereira Figueiredo, Marcelo Resende de Souza, Itallo Conrado Sousa Araujo, Leonardo Jose Camargos Lara

**Affiliations:** Universidade Federal de Minas Gerais, Belo Horizonte, Brazil

**Keywords:** Bacterial identification, MALDI-TOF MS, Newly hatched chick, Ultraviolet light-C irradiation

## Abstract

This study aimed to evaluate the effect of different sanitization procedures on the eggshell surface and yolk sac microbiota of one-day-old chicks. Hatching eggs were collected from a 70-wk-old Cobb500^®^ commercial broiler breeder flock and distributed in a completely randomized design with seven treatments: fumigation with paraformaldehyde (4.63 g/m^3^/30 min; FORM), fumigation with ozone (5-15ppm/30 min; Ozone), ultraviolet light-C irradiation (8.09 mW/cm^2^; 120 s; UV-C), hydrogen peroxide spraying (3%; 0.69 mL/egg; H_2_O_2_), peracetic acid spraying (0.3%; 0.69mL/egg; PAC), water spraying (0.69mL/egg; Water) and without sanitization (dry control - Dry). Culture-dependent methodology was carried out to isolate the microorganisms from eggshell surface (*n* = 224) and yolk sac in one-day-old chicks (*n* = 91), followed by identification by mass spectrometry (MALDI-TOF). Proteobacteria phylum was dominant on egg structures evaluated, with nineteen genera identified on the eggshell surface and twelve in the yolk sac. The most abundant genera found on eggshell surfaces and in yolk sacs were *Staphylococcus* spp. (18%) and *Escherichia* spp. (32.6%), respectively. Among the sanitization protocols tested, ultraviolet light-C irradiation and peracetic acid spraying decreased the presence of *Staphylococcus* spp. and *Escherichia* spp. in hatching eggs. Eggshell and yolk sac microbiota of newly hatched chicks were dominated by *Staphylococcus* and *Escherichia* species, respectively. UV-C irradiation and peracetic acid effectively reduced eggshell *Staphylococcus*, with peracetic acid also decreasing yolk sac *Escherichia*. These findings support UV-C and peracetic acid as effective alternatives to formaldehyde in commercial hatcheries.

**Supplementary Information:**

The online version contains supplementary material available at 10.1007/s42770-026-01896-x.

## Introduction

Sanitization of hatching eggs is a widely procedure applied to minimize the effects of eggshell contamination, such as poor hatchability and decreased chick performance [1, 2]. According to Cadirci [3], fumigation with formaldehyde is the most effective way to sanitize hatching eggs. However, since the publication of the effects of repeated or prolonged exposure to formaldehyde in 1991 by the Occupational Safety and Health Administration (OSHA) [4], several studies have endeavored to find alternative sanitizers. Recent studies have also explored plant-derived compounds as sanitizing agents for hatching eggs. Essential oils such as oregano, thyme, clove, and cinnamon, as well as plant extracts rich in phenolic compounds, have demonstrated antimicrobial activity against eggshell contaminants and have been evaluated as potential alternatives to chemical fumigation. These natural products act mainly through membrane disruption and oxidative stress, reducing bacterial loads on contaminated shells and improving hatchability in some experimental settings [5]. Melo et al. [2] demonstrated the potential for the application of ultraviolet radiation (UV-C) and peracetic acid spraying for eggshell sanitization instead of formaldehyde. Nevertheless, the evaluation of the efficiency of different sanitization procedures has been limited mainly regarding bacterial counts and their impacts on hatching parameters [2, 6–8]. The reduction of microbial load on the eggshell surface is strongly associated with improved hatchability, lower incidence of early embryonic mortality, and better chick quality. Several authors have demonstrated that effective sanitization protocols directly influence incubation outcomes by minimizing contamination-related embryo losses [6].

Contamination of hatcheries by *Escherichia coli* (*E. coli*) is a major concern in poultry farming, particularly affecting the survival and health of chicks in their initial days. *E. coli*, a Gram-negative bacterium, is highly motile due to peritrichous flagella, allowing it to spread easily within poultry environments [9]. This pathogen is a leading cause of bacterial infections globally, with avian pathogenic *E. coli* strains (APEC) causing significant issues in poultry, such as pericarditis, peritonitis, and airsacculitis [10, 11]. The bacteria often enter the hatchery environment via fecal contamination and can infect eggs and newly hatched chicks. Effective monitoring and control of *E. coli* in hatcheries are vital because its presence can lead to decreased hatchability and high mortality rates in chicks, especially during the critical first week of life. Field studies have shown that mortality rates due to *E. coli* infections can be as high as 20%, underscoring the need for stringent biosecurity and hygiene measures to safeguard poultry production [12]. Beyond sanitization, multiple strategies are used to control *E. coli* in poultry production, including vaccination of breeders and broilers, competitive exclusion products that modulate the intestinal microbiota, strict hatchery hygiene programs, improved litter management, and biosecurity-enhancing measures aimed at reducing horizontal transmission [13].

In addition to *E. coli*, other bacteria such as *Salmonella* spp., *Staphylococcus* spp., and *Pseudomonas* spp. also pose significant threats to hatcheries and the early health of chicks. *Salmonella* spp. is particularly notorious for contaminating eggs and causing systemic infections that can lead to both high mortality rates and potential transmission to humans [14, 15]. *Staphylococcus* spp. is correlated with several clinical disorders such as tenosynovitis, omphalitis, femoral head necrosis, infected hock and stifle joints, and pododermatitis. *Staphylococcus aureus* has been recognized as the second most important bacterium accountable for yolk sac infections [16]. Pseudomonas species are associated with respiratory issues that further compromise chick survival. *Pseudomonas aeruginosa* induces a significant economic loss to the farm by causing high mortality of newly hatched chickens and mass death of embryos at later stage [17]. These pathogens highlight the critical need for comprehensive hygiene and monitoring protocols in hatcheries to prevent the spread of infections during the vulnerable early stages of chick development. Control strategies for these pathogens include enhanced breeding flock monitoring, environmental sampling, vaccination programs (particularly against *Salmonella* spp.), water sanitation, air-handling improvements in hatcheries, and the use of disinfectants with proven efficacy against Gram-positive and Gram-negative organisms. Probiotics and bacteriophages have also been investigated as complementary tools for reducing pathogen pressure during the pre-hatching period [18, 19].

The sanitizing agents evaluated in the present study differ substantially in their chemical characteristics and mechanisms of action. Paraformaldehyde acts as a potent alkylating agent capable of inactivating a wide range of microorganisms but poses occupational health risks. Ozone is a strong oxidizing gas with rapid antimicrobial activity but is highly unstable and requires controlled application. UV-C irradiation disrupts microbial DNA and is effective for surface sanitization without chemical residues. Hydrogen peroxide acts through oxidative damage and is commonly used due to its safety and ease of application. Peracetic acid combines oxidative and acidic activity, providing broad-spectrum antimicrobial action with rapid degradation into non-toxic residues. These contrasting properties may influence their effectiveness on eggshell microbiota and subsequent colonization of the yolk sac [20, 21].

Given these risks, it is essential to understand how sanitization protocols affect the microbiotas on the eggshell surface and in the yolk sac at hatching. Thereby, the aim of this study was to evaluate protocols for sanitizing hatching eggs (ozone fumigation, formaldehyde fumigation, ultraviolet radiation, hydrogen peroxide, and peracetic acid spraying) and its impact on the microbiota composition on eggshell surface and in yolk sac of one-day-old chick.

## Materials and methods

### Egg collection

A total of 450 hatching eggs were collected for microbiological analyses and 539 were submitted to incubation, totaling 989 eggs from a 70-wk-old Cobb500^®^ commercial breeder flock. The eggs were originated from the second and third collections of the day. Eggs that were laid overnight and had visible fecal or egg contamination were not used for the experiment. All eggs were collected directly from the nest with disposable gloves. After collection, double-yolked, deformed, or cracked eggs were excluded. The selected eggs were randomly assigned to sanitization treatments, for a total of 65 eggs per treatment. Five sanitizing procedures were employed: fumigation with ozone, fumigation with paraformaldehyde (FORM), radiation with ultraviolet light C (UV-C), spraying with hydrogen peroxide (H_2_O_2_), spraying with peracetic acid (PAC). Two controls were performed: spraying with water (Water) and without sanitizing (Dry). Thus, a total of seven treatments were carried out. The sanitizing procedures were performed in a separate room at the farm after each egg collection.

### Sanitization procedures

#### Ozone

After distributing the eggs among the seven treatments, those designated for the ozone treatment were placed into plastic boxes and transported to a fumigation chamber measuring 8.64 m^3^. Within this chamber, the eggs underwent sanitization using ozone gas, with concentrations ranging from 5 to 15 ppm for 30 min, as per the manufacturer’s instructions (Alvap Engenharia LTDA, Lajeado, Rio Grande do Sul, Brazil), to achieve the desired disinfection levels. The ozone gas was produced by passing compressed oxygen through an ozone generator (Alvap, Lajeado, Rio Grande do Sul, Brazil), while the gas levels within the chamber were monitored using an Ozone Analyzer UV-100 (Eco Sensors, Santa Fe, New Mexico, USA). Throughout the fumigation process, the temperature and relative humidity within the chamber were maintained at 23 to 24 °C and 65 to 70%, respectively. Following fumigation, the ozone concentration within the chamber was reduced to zero through ventilation, with an average total ventilation time of five minutes.

#### Paraformaldehyde

The paraformaldehyde treatment involved placing the eggs in plastic boxes and subjecting them to disinfection within the same chamber (8.64 m^3^) previously used for ozone treatment. As a result, this treatment was implemented one hour later than the others. Fumigation was conducted using a paraformaldehyde concentration of 4.63 g/m^3^, achieved by heating 40 g of paraformaldehyde prills, for 30 min. This was followed by a 10-minute exhaust period before opening the chamber. The setup ensured proper circulation of the gas throughout the chamber.

#### UV-C irradiation

The UV-C treatment was performed following Melo et al. [2], with 16 germicidal UV-C lamps (30 W, 90 cm, 254 nm, HALOTECH). Eggs were placed in a 90-egg custom-built metal wire flat that was designed to minimize contact between the egg and the wire. The eggs were exposed to UV light at a mean intensity of 8.09 mW/cm^2^ for 120 s. The UV intensity was measured by a probe (THORLABS S401C, Dachau, Germany) coupled to a digital optical power meter (THORLABS PM100D, Dachau, Germany).

#### Hydrogen peroxide

The H_2_O_2_ treatment was applied to eggs by spraying a fine mist of 3% (1,280 ppm) H_2_O_2_ aqueous solution (Oxivir Five 16 Concentrate, Diversey, Socorro, São Paulo, Brazil) using a hand-held manual sprayer (0.69 mL/egg). The temperature of the solution was recorded by a digital thermometer (Incoterm 5004, São Paulo, São Paulo, Brazil) and was set at 21.5 °C. To cover the entire surface of the eggs with the solution, the trays with the eggs were placed on a flat surface and sprayed in two steps: half the volume of the disinfectant solution was administered on one side of the eggs, after which the eggs were turned manually, and the remainder of the solution was applied to the other surface.

#### Peracetic acid

The PAC treatment was applied to eggs by spraying a fine mist of 0.3% PAC (Divosan Forte, Diversey, Socorro, São Paulo, Brazil) using a hand-held manual sprayer. The solution was prepared immediately prior to use by mixing the commercial product with water according to the directions of the manufacturer to achieve the desired concentrations. The spray mode and volume were the same those used in the hydrogen peroxide treatment. The temperature of the solution was recorded by a digital thermometer (Incoterm 5004, São Paulo, São Paulo, Brazil) and ranged from 19.5 °C to 20.5 °C.

#### Without sanitization (water control)

The same protocol used for spraying eggs with H_2_O_2_ and PAC was applied to the eggs of this treatment, although they were sprayed with water. The purpose of this treatment was to detect any mechanical effects that could occur from the washing process. The temperature of the water was recorded by a digital thermometer (Incoterm^®^5004, São Paulo, São Paulo, Brazil) and ranged from 19.5 °C to 20.5 °C.

#### Without sanitization (dry control)

The eggs of this treatment were kept in the same room in which the other treatments were placed, but they were not submitted to any disinfection procedure or sprayed with water.

### Incubation

One day after sanitization, the 989 hatching eggs were transported to the commercial hatchery. During this storage period, the eggs remained at a temperature from 22 to 24 °C and humidity from 55 to 60% overnight at the breeder farm. The eggs were placed in trays, one per treatment, with a capacity of 77 eggs each. The eggs from the seven treatments were incubated together at 37.4 °C (99.3 °F) and Relative Humidity (RH) set at 62% (84 °F) in a multi-stage setter (Casp CMG 125E, Amparo, São Paulo, Brazil), with a capacity of 124,416 eggs. Trays were put in a trolley and incubated. Eggs were turned at an angle of 45° at a frequency of 24 times a day. On the 14th day of incubation, all eggs were candled to remove the infertile ones and those with early embryonic mortality. The eggs were transferred from the setter to the hatcher (CASP 21E, Amparo, São Paulo, Brazil) after 456 h of incubation (19 days).

The eggs were transferred from the trays to hatcher baskets, which were labeled according to treatments. All hatcher baskets were randomly distributed inside the hatchery and kept at an average temperature and an RH of 36.6 °C (98 °F) and 65%, respectively. After 504 h of incubation, all hatched chicks were removed from the hatcher. Chick quality was visually assessed and chicks deemed not saleable according to the commercial hatchery standards (with unhealed navels, red hocks, or obvious abnormalities) were recorded. Unhatched eggs were counted, opened and examined macroscopically to determine infertility percentage, percentage of embryonic mortality [early (0 to 7 d), middle (8 to 14 d), and late (15 to 21 d plus pipped)], and hatchability percentage of fertile and total eggs, and to record observations of cracked (data not shown) and contaminated eggs. No disinfection was performed in the incubator or in the hatcher during the experimental period.

### Bacterial isolation

#### Eggshell surface

One hour after sanitization, 16 eggs per treatment per collection were sampled for eggshell microbiological analyses, totaling 32 eggs per treatment. The eggs, collected with disposable gloves, were placed in sterile plastic bags (four eggs per bag) and refrigerated at 4 °C until being transported to the laboratory (microbiological analyses were performed 24 h after sampling). Each bag containing a pool of four eggs was aseptically reopened and the eggs were transferred to another sterile plastic bag, in which 250 mL of sterile phosphate-buffered saline (PBS) was added. A rinse sample was obtained by massaging the bag for five minutes to remove bacterial cells from the surface of the eggshells. One mL of the resultant rinse solution was diluted serially using PBS. Then, aliquots of 100 µL were inoculated on Plate Count Agar (PCA) (Oxoid LTD., Basingstoke, Hampshire, England) and MacConkey (BD Difcotm, Sparks, Maryland) for isolation. The plates were incubated at 37 °C for 48 h in order to allow bacterial growth.

#### Yolk sac

A total of 13 saleable chicks per treatment were randomly chosen and transported to the laboratory for microbiological analyses. At 24 h post-hatching, the chicks were euthanized by cervical dislocation to aseptically collect the yolk sac. One gram of the yolk content per chick was sampled for serial dilution in PBS. A 100 µl aliquot was inoculated on Plate Count Agar (PCA) and MacConkey Agar for bacterial growth. All plates were incubated at 37 °C for 48 h.

### Bacterial identification

For the determination of the microbiota composition of eggshell surface and yolk sac from chicks at 24 h post-hatching, the Matrix-Assisted Laser Desorption Ionization – Time of Flight Mass Spectrometry (MALDI-TOF MS) technique was applied. A total of 396 colonies with different morphological and pigmentation characteristics were picked from each plate (PCA and MacConkey Agars), diluted and dispersed in a matrix (organic acid). Colonies were selected visually based on distinct morphology, pigmentation, and growth characteristics to ensure representative diversity from each plate. Each sample was placed on a specific plate for this analysis. The purification was performed by inoculating each colony in 5 mL Infusion Brain Heart (BHI) broth and incubating for 24 h at 35 °C ± 2 °C. Then, aliquots from BHI broth were streaked onto BHI agar and incubated for 20 to 24 h at 35 °C ± 2 °C, to obtain pure colonies.

A fresh, single colony of each bacterial isolate was spotted using a toothpick into a target steel plate. For each strain, 1 µl of formic acid (70%) and 1 µl of MALDI-TOF MS matrix, consisting of a saturated solution of a-cyano-4-hydroxycinnamiccid (HCCA) (Bruker Daltonics, Bremen, Germany), were applied to the spot and allowed to air-dry. Spectra were acquired using the FlexControl MicroFlex LT mass spectrometer (Bruker Daltonics). Prior to measurements, calibration was preceded with a bacterial test standard (*E. coli* DH5 alpha; Bruker Daltonics). The Real Time (RT) identification score criteria used were those recommended by the manufacturer: ≥ 2,000 indicates a species-level identification, ≥ 1,700 and < 2,000 a genus-level identification, and < 1,700 indicates no reliable identification target for each run [22].

### Data analysisf

A completely randomized experimental design was applied specifically to define the number of replicates per treatment for the bacterial isolation procedures. Eggshell samples consisted of eight replicates, each formed by a pool of four eggs, whereas yolk sac samples consisted of 13 individual replicates per treatment. The term “randomized” refers to the random allocation of eggs to the replicates within each treatment. After isolation and identification, data were analyzed and interpreted using descriptive statistics (Excel Microsoft).

## Results

### Bacterial identification on eggshell surface

**Fig. 1:**
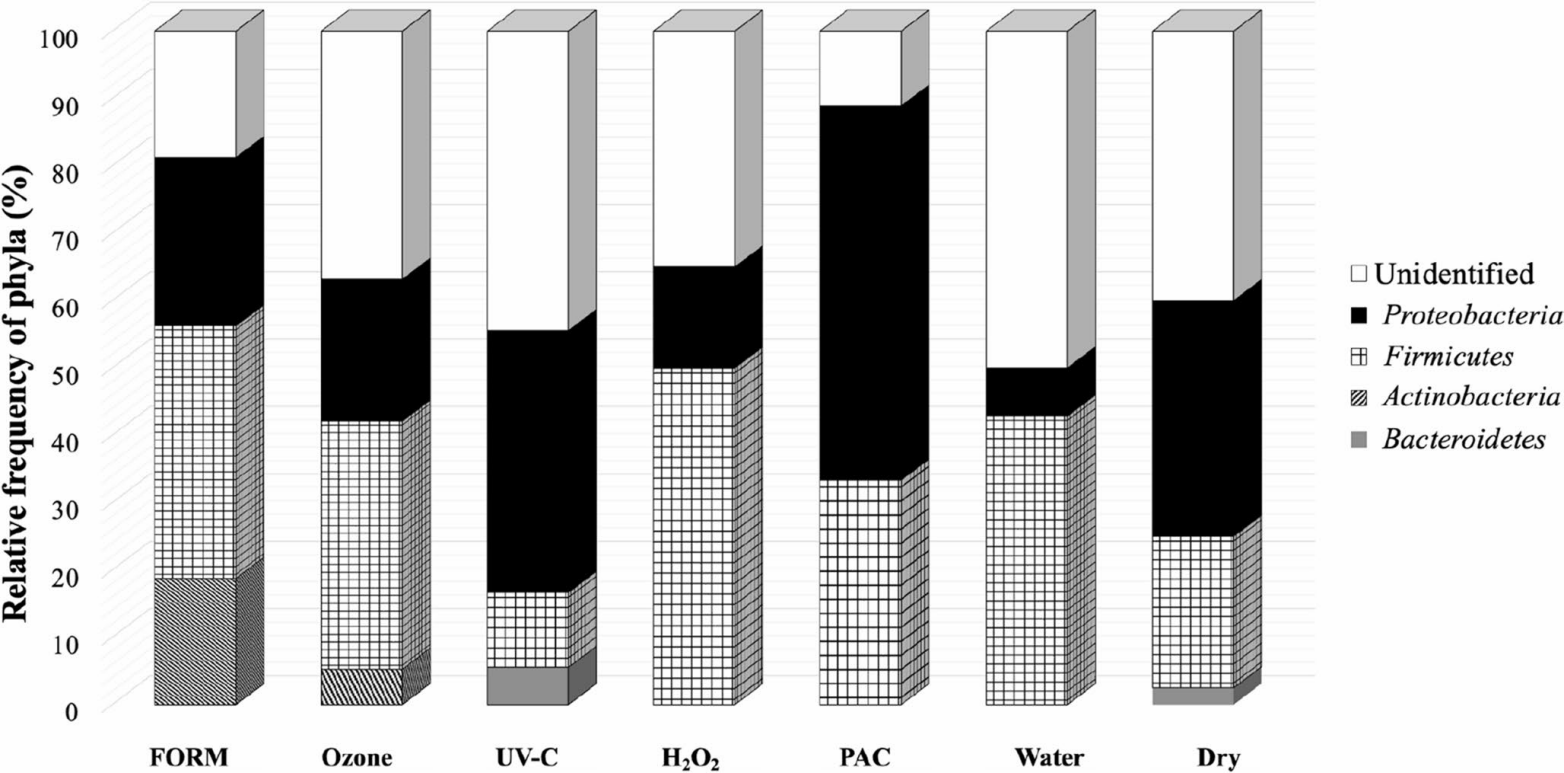
The relative frequency of phyla detected on chicken eggshell one hour after different sanitization procedures

A total of four phyla were found on eggshell surface (Fig. 1). Proteobacteria and Firmicutes were the two most dominant, although the phyla distribution among the treatments was variable. Actinobacteria was detected only in FORM and Ozone treatments, with 30.0 and 7.1% of relative frequency, respectively. Bacteroidetes was found in the UV-C (7.8%) and the Dry (3.2%) treatments. Firmicutes was present in H_2_O_2_ and PAC treatments, with 40.1 and 37.5%, respectively. Proteobacteria presented higher relative frequency in PAC treatment (50.0%) when compared with all others (FORM, 30.0%; Ozone, 21.4%; UV-C, 31.0%; H_2_O_2_, 13.4%; Water, 11.1% and Dry, 25.7%). Phylum-level classification was obtained by mapping MALDI-TOF-identified genera to their corresponding higher taxonomic ranks using the LPSN and NCBI Taxonomy databases.

**Fig. 2:**
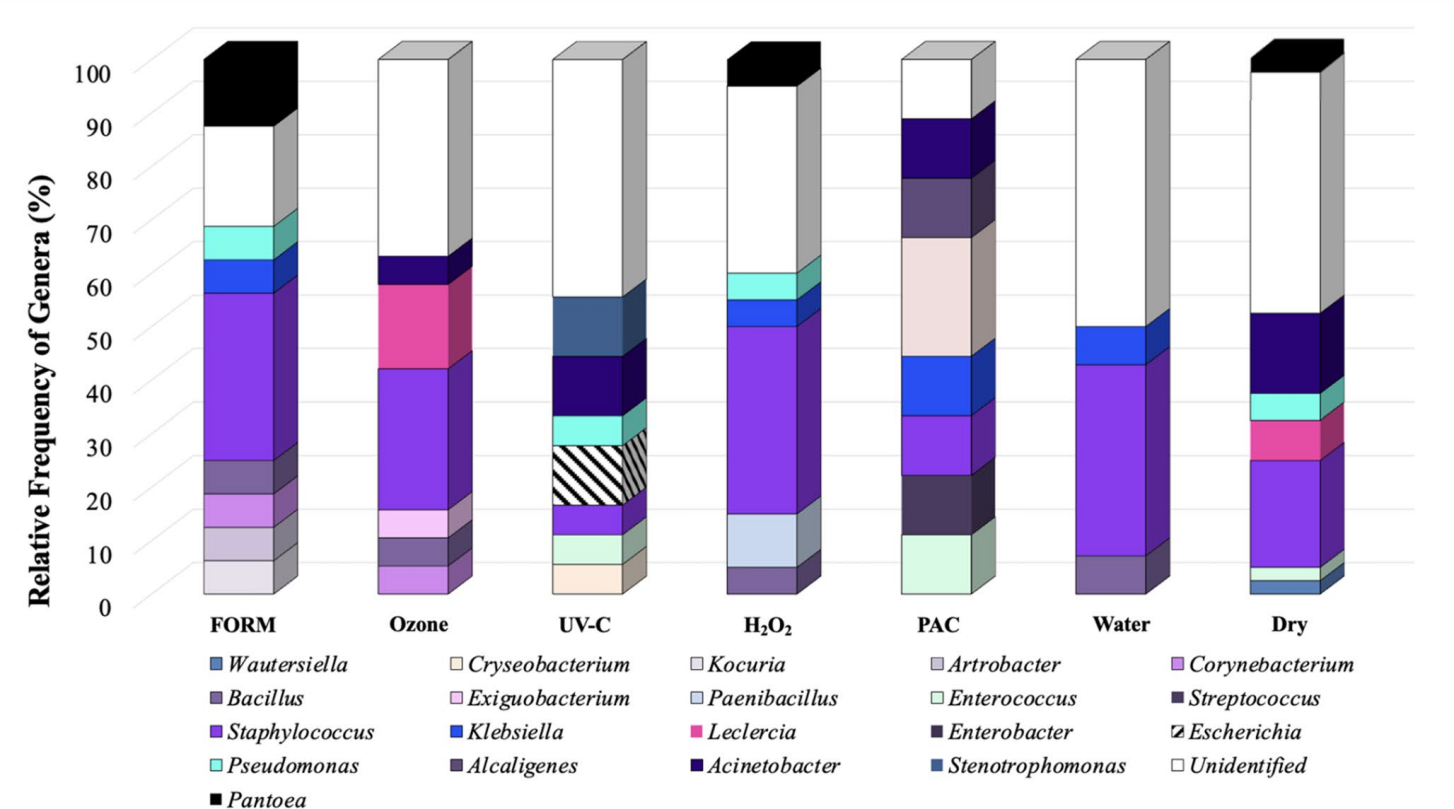
The relative frequency of genera detected on chicken eggshell one hour after different sanitization procedures

MALDI-TOF MS allowed identifying a total of 20 bacteria genera (Fig. 2). *Staphylococcus* spp. was the dominant bacterium isolated from eggshell (Fig. 2). The treatment Water presented approximately 52% of Unidentified Genera, which was also observed in Ozone (50.0%), UV-C (53.4%), H_2_O_2_ (46.7%) and Dry (58.1%). Unidentified Genera in PAC group was 12.5%. FORM group presented seven different genera on eggshell surface (*Kocuria*,* Corynebacterium*,* Arthrobacter*,* Bacilus*,* Pantoea*,* Pseudomonas and Klebsiella*) with 10% of relative frequency each. *Escherichia* was identified only in treatment UV-C (15.4%).

In our experiment, Gram-negative and Gram-positive bacteria were found in almost the same frequencies: 54% and 46% respectively. Only *Pseudomonas* (treatments FORM, UV-C, H_2_O_2_ and Dry control) and *Alcaligenes* were isolated in low relative frequency, which ranged from 1.16 to 5.81% of the total bacteria isolated (Fig. 2).

### Bacterial identification in yolk sac of newly hatched chicks

**Fig. 3:**
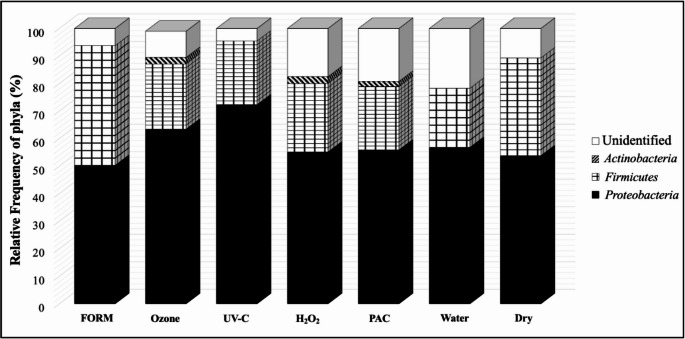
The relative frequency of phyla detected in yolk sac of newly hatched chicks, according to the different sanitizers used on the eggshell

In the yolk sac of one-day-old chicks, three phyla were identified: Proteobacteria (67.4%), Firmicutes (31.3%) and Actinobacteria (3.0%) (Fig. 3). Actinobacteria was only found in the Ozone, H_2_O_2_, and PAC treatments, constituting 2.4%, 2.5%, and 1.9%, respectively. *Proteobacteria* had the highest frequency in all treatments when compared to other phyla found. In the UV-C treatment, 72.1% of the microorganisms belonged to that phylum, while in the H_2_O_2_, Ozone, PAC, Water and Dry control treatments, the frequency of *Proteobacteria* was about 55% each. Firmicutes was found in greater quantity in the yolk sacs of chicks from eggs sanitized with formaldehyde (43.75%), followed by unsanitized eggs (35.72%), H_2_O_2_ (25%), Ozone (23.81%), UV-C (23.26%), PAC (23.07%) and Water (21.74%).

**Fig. 4:**
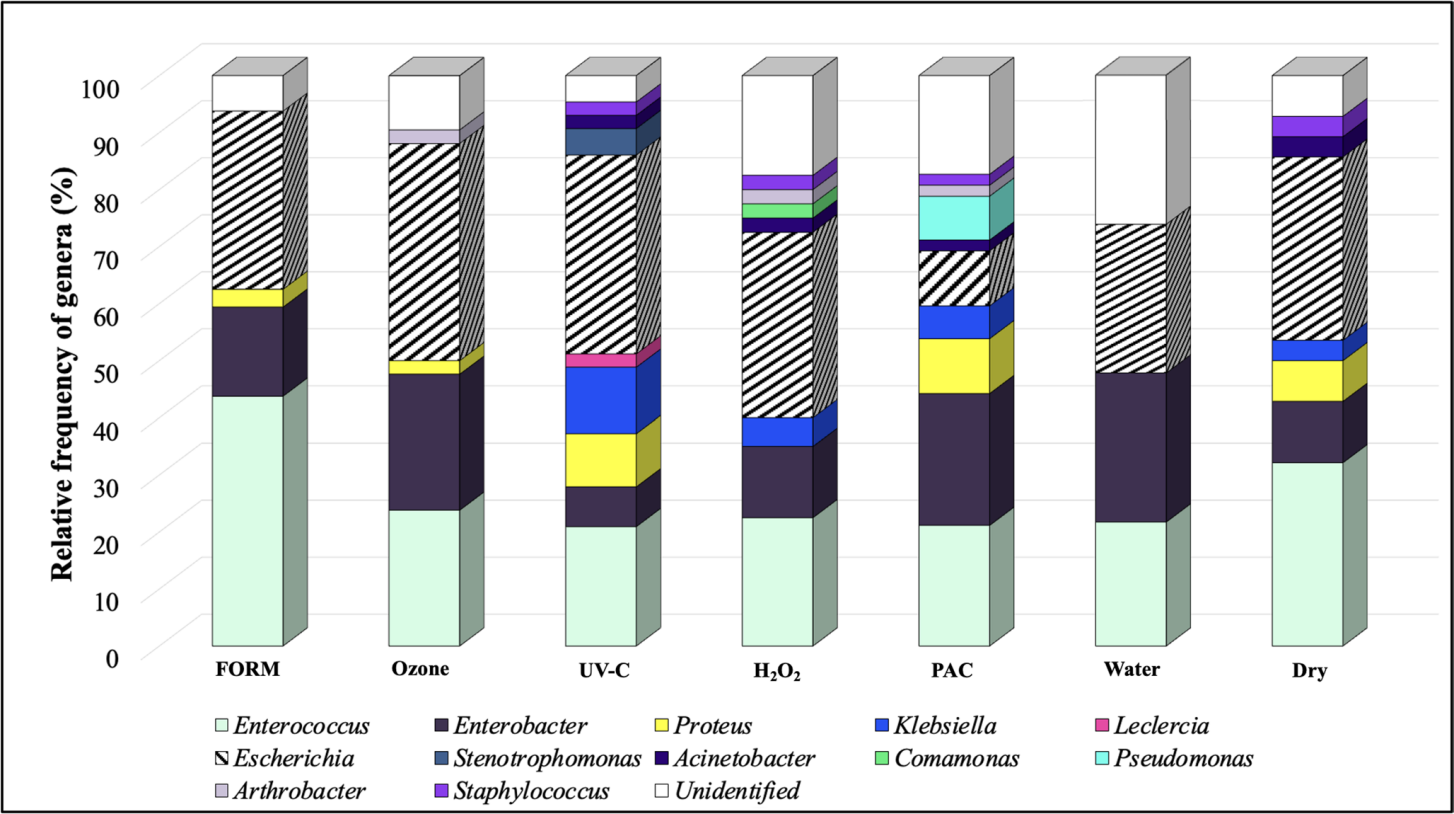
The relative frequency of genera detected in yolk sac of newly hatched chicks, according to the different sanitizers used on the eggshell

A total of 12 genera were identified in yolk sac of newly hatched chicks, with *Escherichia* (32.6%), *Enterococcus* (29.5%) and *Enterobacter* (19.4%) being the most dominant considering the different treatments, as shown in Fig. 4. The Water treatment exhibited the highest percentage of unidentified genera (29.73%), followed by PAC (19.23%), H_2_O_2_ (17.5%) and Dry control group (10.71%). In the yolk sac of chicks from FORM treatment, *Enterococcus* was the most prevalent genus (43.75%), followed by *Escherichia* (31.25%) and *Enterobacter* (15.62%). Notably, *Enterococcus*,* Acinetobacter* and *Escherichia* were consistently detected in all treatments (Fig. 4). The UV-C treatment showed a lower frequency of *Enterobacter* (6.98%) compared to the Water (26.09%) and Dry control (10.71%) treatments.

The PAC treatment had the lowest frequency of *Escherichia* (9.62%) when compared to the other sanitizers (*Escherichia* frequencies: 31.25% in FORM, 38.1% in Ozone, 34.88% in UV-C, 32.50% in H_2_O_2_, 26.09% in Water and 32.15% in Dry control treatments). However, *Pseudomonas* was exclusively found in the PAC, with a frequency of 7.69%.

MALDI-TOF MS identified bacteria at genus level in 87.3% (227/260) of all isolates. For bacteria on eggshell surface, that technique was able to identify 63.7% (86/135). *Enterococcus faecalis* (57/67; 85.1%) was the main *Enterococcus* species isolated. The others *Enterococcus* species isolated were: *E. gallinarum* (6/67; 8.9%), *E. casselifavus* (3/67; 4.5%) and *E. avium* (1/67; 1.5%). The two *Enterobacter* species isolated from yolk sac were *E. cloacae* (38/44; 86.4%) and *E. asburiae* (6/44; 13.6%).

## Discussion

The present study is the first to identify the microbiota of eggshells after the use of paraformaldehyde and alternative sanitization, as well as correlate the protocols of eggshell sanitization for fertile eggs and characterize the microbiota of the yolk sac of newly hatched chicks. Overall, the eggshell microbiota was dominated by bacteria belonging to the phyla Firmicutes and Proteobacteria. Olsen et al. [1] evaluated the microbiome of eggshell surface of visibly clean and dirty eggs, before and after sanitization with paraformaldehyde and reported the dominance of bacteria belonging to the phyla Firmicutes. Maki et al. [23] evaluated the eggshell microbiota at different incubation times, of eggs that were not subjected to pre-incubation paraformaldehyde sanitization. They identified bacteria from the phyla Firmicutes as the most abundant, which was not different compared to the results found in this study.

When comparing eggshell and yolk sac profiles, a clear correspondence was observed between the reduction of specific genera on the eggshell and their presence in the yolk sac. UV-C and PAC, which reduced *Staphylococcus* spp. and overall Gram-positive counts on the shell, also showed lower recovery of Staphylococcus-related genera in yolk sac samples. Similarly, PAC, the only treatment that substantially reduced *Escherichia* spp. on the eggshell, resulted in the lowest *Escherichia* frequencies in yolk sacs. These findings support the hypothesis that the initial shell microbiota strongly influences early yolk sac colonization.

Eggs are formed in an environment that cannot be considered sterile. The reproductive tract of chickens harbors a rich microbiota, including some pathogenic bacteria. Eggs can be directly contaminated with microorganisms during egg formation and oviposition. In addition, some of the microorganisms can be transmitted vertically to the embryo through the egg and constitute the early gut microbiota of the chick [24]. The composition of this microbiota can reflect the health status of laying chicken [1].

Any surface post-laying can be the source of eggshell contamination. Eggs may become contaminated during their production, storage and transportation. For example, *Salmonella* spp. and *E. coli* can penetrate eggshells from contaminated feces and the environment during or after laying. *Salmonella* spp. can also originate from infected reproductive organs and contaminate eggs before the formation of the shell. *Staphylococcus aureus* contamination in eggshells may also occur from the handler and the environment during egg storage and transportation [25]. This is the reason that is so important to maintain high standards of hygiene at the farm, to avoid eggshell contamination (after laying) and re-contamination (after sanitization process).

Higenyi and Kabasa [26] reported the prevalence of several microorganisms on the eggshell surface, including *Escherichia coli* (19%), fungi (3%), *Proteus* spp. (2%), *Pseudomonas aeruginosa* (9%), and *Staphylococcus aureus* (18%), while *P. aeruginosa* (4%) and *S. aureus* (4%) were isolated from the internal contents of eggs. Similarly, Verma et al. [27] identified *E. coli* and *Staphylococcus* spp. on chicken eggshell samples. These findings demonstrate that eggshell-associated microbiota can vary substantially depending on environmental and sanitary conditions. In the present study, differences in microbial composition among treatments indicate that each sanitization protocol modifies the eggshell environment and may influence microbial penetration during incubation. Treatments based on oxidative mechanisms (H₂O₂ and peracetic acid) tended to reduce Gram-positive genera, whereas UV-C irradiation and peracetic acid showed broader effects, decreasing both Firmicutes and Proteobacteria. This pattern is consistent with previous reports indicating that sanitizers with multiple modes of action promote wider microbial suppression.

On the other hand, Petrovič et al. [28] collected samples at the broiler breeder farms and *Staphylococcus* spp. was the main bacterium isolated from the nest material. They found that the most isolated species were *Staphylococcus equorum* (8%) and *Staphylococcus equorum* subsp. *equorum* (6%). The other most isolated bacterial species were *Ralstonia picketii* and *Staphylococcus haemoliticus* (6%) and *Ralstonia mannitolilytica* and *Pseudomonas luteola* (5%). These results could explain why this genus is the most prevalent bacterium isolated from eggshell surface since all the samples were collected directly from the nest.

The microbiota of the chicken eggshell is dominated by Gram-positive bacteria [29, 30], which may be related to the contamination sources post-laying. In our experiment, Gram-negative and Gram-positive bacteria were found in almost the same frequencies on eggshell surface: 54% and 46% respectively. According to Varghese and Balachandran [31], Gram-negative bacteria are less susceptible to antibiotics and more pathogenic. More important than identify all bacteria on the eggshell surface is to identify the ones that are involved with spoilage. The most common microorganisms isolated from rotten eggs are members of the genera *Alcaligenes*, *Pseudomonas*, *Proteus* and *Aeromonas* [30]. Regarding the relative frequency of genera, although water protocol does not have antimicrobial activity, its mechanical effect may reduce the number of loosely attached microorganisms on the eggshell surface, thereby limiting the diversity of bacteria able to reach and colonize the yolk sac. In addition, the absence of selective antimicrobial pressure may favor the dominance of a limited number of taxa, resulting in lower microbial diversity compared with chemical or physical sanitization methods.

Bacteria present on the eggshell surface may penetrate the egg through shell pores, especially when a temperature differential exists between the egg and the surrounding environment [30]. Such bacterial penetration can result in embryonic contamination, reduced hatchability, and an increased incidence of rotten eggs during incubation. According to Araújo et al. [32], contaminated eggs are frequently identified during residual analyses of unhatched eggs, representing a major challenge for hatcheries, particularly when eggs originate from breeder flocks older than 65 weeks. Furthermore, contamination of eggs by gas-producing bacteria may cause internal pressure buildup, leading to shell cracking and subsequent widespread contamination of neighboring eggs in setters and hatchers.

Similar findings were obtained by Karunarathna et al. [33], who isolated bacteria from unhatched broiler eggs from two commercial hatcheries and subsequently identified the isolates by mass spectrometry, with 83.1% being classified at the genus level. These results demonstrate the potential of MALDI-TOF MS technique to identify bacteria in different circumstances. Probably because the database used includes spectra from relevant bacteria for human and veterinary medicines, leaving aside bacteria present in the environment. According to Holl et al. [34], depending on the quality and the quantity of the reference mass spectrometry profiles, the identification of unknown bacteria is not possible, and the result occurs as not reliable identification.

*Escherichia coli* is the most common bacterium isolated from chicks with yolk sac infection and is a major cause of mortality in broilers during the first week [35]. This bacterial infection leads to acute and chronic diseases during the entire growth period of broiler chickens, including increased mortality due to septicemia in neonatal chickens, chronic joint diseases, poor performance, poor feed conversion efficiency, loss of uniformity of the flock, and downgrading and increased condemnations at processing. Karunarathna et al. [33] demonstrated by MALDI-TOF MS analysis that the majority of nonviable eggs presented contamination by *Enterococcus* species (29.7%), mainly *E. faecalis*, followed by *E. coli* (19.5%). These results are in agreement with the findings of this study.

The dominance of *E. faecalis* in the yolk sac in our study could be explained by the results of Saad et al. [36]. *E. faecalis* is part of the normal intestinal microbiome of animals and humans and it has been found to be among the dominating intestinal of day-old chicks. However, *E. faecalis* is also considered an opportunistic pathogen with the potential to cause clinical infections. In chickens, it is often associated with septicemia, endocarditis, and salpingitis, and may subsequently lead to amyloid arthropathy and amyloidosis. *E. faecalis* affects avian species of all ages; however, the majority of serious infections have been associated with embryos and young birds.

Chicks are particularly susceptible to infections during the first week of life and substantial mortality may occur due to omphalitis (yolk sac infection) and septicemia. Olsen et al. [37] investigated the transmission of *E. faecalis* during of hatching of broiler chicks. They found that 15% of *E. faecalis* infection is transmitted vertically and this transmission is five times greater when the parent flocks were older than 42 weeks. According to Eyasu et al. [38], bacteria isolated from poultry litter in five farms showed *Enterobacter cloacae* in 4% of all collected samples. In the present study, the relative frequency of *Enterobacter* across treatments was higher, ranging from 6.98% to 26.09%. Moreover, characterizing the microbiota of unhatched chicks and hatching eggs is essential for identifying bacterial taxa potentially associated with reduced hatchability and compromised chick quality.

The predominance of *Enterococcus* spp. and *Escherichia* spp. in the yolk sac microbiota observed in this study is consistent with the findings of Vieira et al. [39], who identified these genera as major agents associated with yolk sac infection and high early chick mortality. In our results, *Enterococcus* spp., mainly *Enterococcus faecalis*, were detected across all sanitization protocols, indicating that early colonization may occur during incubation or shortly after hatching, regardless of eggshell treatment. Vieira et al. [39] reported that infections caused by *E. faecalis* and *E. coli* were the primary contributors to first-week mortality, even in chicks with healed navels, supporting our observation of widespread yolk sac colonization by these bacteria. Together, these findings reinforce the opportunistic pathogenic role of *Enterococcus* spp. during the neonatal period and highlight the importance of eggshell sanitization strategies capable of limiting the early transfer of these microorganisms to improve chick health outcomes.

From an industrial perspective, the feasibility of these protocols depends on cost, required infrastructure, exposure uniformity, and operator safety. UV-C and PAC, which were among the most effective in this study, are compatible with automated systems and generate no harmful residues, making them suitable for large-scale hatcheries. Ozone and hydrogen peroxide require more controlled application but remain viable alternatives depending on equipment availability. For future studies, it is important to investigate the efficiency of sanitizers against relevant bacteria for the poultry industry to determine the best antimicrobial agent in each situation.

## Conclusions

This study demonstrated that the eggshell surface and yolk sac microbiota of newly hatched chicks are dominated by *Staphylococcus* and *Escherichia*, respectively. Among the evaluated sanitization protocols, UV-C irradiation and peracetic acid spraying were the most effective in reducing *Staphylococcus* spp. on the eggshell, while PAC was also associated with a marked decrease in *Escherichia* spp. in yolk sacs. These findings highlight the direct relationship between eggshell microbial reduction and early yolk sac colonization. Considering their efficacy and practical applicability, UV-C and PAC represent promising alternatives to formaldehyde for use in commercial hatcheries.

## Supplementary Information

Below is the link to the electronic supplementary material.

Supplementary Material 1

## Data Availability

Not applicable.
