# Supplementary material for: Alternative protocols for sanitizing hatching eggs and their effects on the microbiota of eggshell surface and chick yolk sac
Source: Braz J Microbiol. 2026 Mar 6;57(1):72. doi: 10.1007/s42770-026-01896-x (PMC12963590; doi:10.1007/s42770-026-01896-x)
Supplement: Supplementary file 1 — Supplementary Material 1 [file 42770_2026_1896_MOESM1_ESM.docx]

Belo Horizonte, Jan 05 2025.

Manuscript ID: BJMI-D-24-01235

We are pleased to submit the second revised version of our manuscript entitled “An evaluation of alternative protocols for sanitizing hatching eggs on eggshell and yolk sac microbiota”. All changes made in response to the reviewers’ comments are highlighted in yellow in the revised manuscript.

Substantial revisions were carried out throughout the **Introduction, Methodology, and Discussion** sections. We carefully addressed the comments and suggestions provided by all four reviewers, which significantly contributed to improving the clarity, scientific rigor, and overall quality of the manuscript.

We sincerely thank the editors and reviewers for their constructive and insightful feedback. We believe that the manuscript has been considerably strengthened as a result of these revisions and is now more suitable for publication. We remain at your disposal to address any additional questions or comments, should they arise.

Yours sincerely,

Authors.

Recommendation: Major Revision

**RESPONSES TO THE COMMENTS OF REVIEWER 1**

Comments:

Replace the word "methods" with "protocols" in the title and throughout the text.

RESPONSE: Done as suggested in all paper.

Line 29 - Cite additional products that have been tested before mentioning the study by Melo et al. The use of natural plant compounds has been extensively investigated, such as natural extracts and essential oils. Within the context of hatching egg sanitization, have these products also been tested? Cite them.

RESPONSE: Done as suggested. L. 33-38.

Line 32 - Discuss the correlation between sanitization, bacterial counts, and incubation parameters.
RESPONSE: Done as suggested. A new sentence was added linking the impact of sanitization protocols on eggshell microbial load, hatchability, and chick quality, with supporting reference. L. 41-45.

Line 42 - In addition to sanitization, mention other approaches for controlling *E. coli* in poultry production.

RESPONSE: Done as suggested. We added examples of additional control strategies such as vaccination, competitive exclusion, improvements in farm hygiene, and antimicrobial stewardship programs. L. 56-59.

Line 53 - Also cite other control strategies for the bacteria discussed in the paragraph.
RESPONSE: Done as suggested. We added examples of additional control strategies such as vaccination, competitive exclusion, improvements in farm hygiene, and antimicrobial stewardship programs.

After line 53, add a new paragraph describing the characteristics of the products evaluated in the study.

RESPONSE: Done as suggested. A new paragraph has been inserted summarizing the chemical nature, mechanism of action, and main application characteristics of ozone, paraformaldehyde, UV-C, hydrogen peroxide, and peracetic acid.

In the "Data analysis" section, specify the programs used in the study.

RESPONSE: Done as suggested. L. 212.

It is recommended that the authors merge the results and discussion sections to make the text more cohesive. The discussion should be improved. For example, what is the relationship between the bacteria identified on the eggshell in the present study and the results of bacterial count analyses (specific bacteria, mesophilic bacteria, Enterobacteriaceae, among others) in eggshells and yolk sacs reported in other studies that evaluated these products as egg sanitizers? Discuss this for all evaluated products.
RESPONSE: We thank the reviewer for the comments and suggestions. However, we respectfully chose **not to merge the Results and Discussion sections**, as we believe that presenting them separately improves clarity and readability of the manuscript. In addition, the journal guidelines allow this structure, and therefore we do not see a need to combine these sections. The Discussion was revised and strengthened to better relate the bacteria identified on the eggshell in the present study with bacterial count data (e.g., mesophilic bacteria and Enterobacteriaceae) reported in previous studies evaluating egg sanitizers. These relationships are now discussed for all evaluated products.

Also discuss the relationship between the application of different sanitizing products on the eggshell and the bacterial counts or identification in the yolk sac.

RESPONSE: Done as suggested. This relationship has now been addressed in the Discussion, comparing the reduction of specific genera on the eggshell with their detection frequencies in yolk sacs.

The conclusion needs to be rewritten to focus more on the key findings obtained in this study regarding the tested products, rather than only suggesting future research.

RESPONSE: Done as suggested.

**RESPONSES TO THE COMMENTS OF REVIEWER 2**

The study evaluated the effect of different sanitization treatments on the eggshell surface and yolk sac microbiota. A culture-dependent methodology was used for bacterial isolation, followed by identification through MALDI-TOF. The results revealed the main genera present on eggshell surface and yolk sac of one-day-old chicks, allowing a comparison among the treatments.

Abstract

Lines 16-18: In the sentence "Among the sanitization methods tested, ultraviolet light-C irradiation and peracetic acid spraying decreased the presence of Staphylococcus spp. and Escherichia spp. in hatching eggs". The sentence is too broad. Lower relative frequency of *Staphylococcus* spp. is clear on eggshell "previously submitted to" UV-C and PAC treatments (Figure 2) and lower relative frequency of Escherichia can be observed in yolk sac of newly hatched chicks from eggs subjected to PAC treatment.

RESPONSE: We thank the reviewer for the comment. The statement in the Abstract was intentionally kept broad, as abstracts are meant to provide a general overview of the main findings, while detailed and specific information is presented in the Results and Discussion sections. In addition, the journal guidelines **do not recommend citing tables or figures in the Abstract**. Therefore, the specific distinctions regarding the relative frequencies of Staphylococcus spp. and Escherichia spp. are appropriately detailed in the main text rather than in the Abstract.

Material and Methods

Bacterial isolation: To better understand the workflow and the number of samples subjected to microbiological analysis, the authors could add a figure illustrating the treatments and timepoints when samples were obtained for analysis.

RESPONSE: We thank the reviewer for the suggestion. However, in our view, the inclusion of an additional figure illustrating treatments and sampling timepoints would not substantially enrich the manuscript. The workflow, microbiological isolation procedures, and the number of samples analyzed are clearly and comprehensively described in the Materials and Methods section, allowing full understanding of the experimental design without the need for an additional schematic figure.

Line 117: PAC instead PAA

RESPONSE: Done as suggested in all document.

Results

The figures 1 and 2 shown relative frequency of phyla and genera detected on eggshell previously sanitization procedures, is that? On lines 146-147 the authors mentioned that eggs were collected for microbiological analysis one hour after sanitization…

RESPONSE: Dear Reviewer 2, We agree that the original wording could lead to misinterpretation. The figure legends were revised to clearly indicate that eggshell microbiota was analyzed after the sanitization procedures (one hour post-sanitization).

Check in the Figure 2 the relative frequency collum of "Dry" treatment and in the Figure 4 the relative frequency collum of "Water" treatment (they looks different, not reaching 100%).

RESPONSE: This was an oversight, and Figure 2 has been corrected accordingly. We thank the reviewer for pointing this out.

Discussion

Lines 257-258: Check the use of "comma" throughout the sentence.

RESPONSE: Done as suggested. Sentence structure and punctuation were revised.

Lines 259-263: Looks like more a results description than results discussion.

RESPONSE: Done as suggested. This section was rewritten to provide interpretation rather than description.

Lines 267-268: Check the use of "comma" throughout the sentence.

RESPONSE: Done as suggested. Sentence structure and punctuation were revised.

Lines 293-295: Redundant sentence.

RESPONSE: The repeated information was removed.

Lines 313-317: Improve this sentence.

RESPONSE: The sentence was restructured for clarity and conciseness.

**RESPONSES TO THE COMMENTS OF REVIEWER 3**

The introduction briefly touches on the sanitization methods used for eggs, addressing them only in the first paragraph. The remaining paragraphs discuss microorganisms and the consequences of their presence on the eggshell. I suggest that the authors expand a little more on the advantages and disadvantages of commercially used egg sanitization methods in the first paragraph.

RESPONSE: Done as suggested. A new paragraph has been added summarizing the operational, economic, and biosafety advantages and drawbacks of formaldehyde, ozone, UV-C, hydrogen peroxide, and peracetic acid.

What does the acronym PAA mean in line 117 of topic 2.2.6. Without sanitization (water control)?

RESPONSE: The acronym PAA in line 117 was a typographical error. The correct acronym is PAC, referring to peracetic acid. This mistake has been corrected in the text, and we appreciate the reviewer’s comment.

The four figures present the same graphic profile. Can the data not be presented in another format or table?  Because they become repetitive when presented in the same format.

RESPONSE: We thank the reviewer for the suggestion. We chose to present the data as graphs because this format facilitates visual comparison among treatments and improves readability and engagement for the reader. In addition, the mean values are already reported in the Results section, avoiding unnecessary redundancy. We believe that maintaining the graphical presentation enhances clarity and draws attention to the main patterns observed in the data.

The authors mention in topic 2.6 that "A completely randomized experimental design was used for bacterial isolation of eggshell and yolk sac, with eight (pool of four eggs) and 13 replicates, respectively, per treatment." It is unclear whether an experimental design was used or if the term "randomized" means that the selection of eggs was done randomly. I suggest that this information be further explained.

RESPONSE: Done as suggested. Lines 213-217.

The conclusion is quite brief. The authors could have cited which microorganisms were most prevalent and which methods were most efficient, but they failed to finalize the article's context by mentioning, for example, whether such methods are applicable in industry or by discussing the cost/efficiency/practical feasibility of their use.

RESPONSE: Done as suggested.

**RESPONSES TO THE COMMENTS OF REVIEWER 4**

The work is very interesting and relevant to the poultry industry. However, the authors need to make some adjustments to improve it.

42. I suggest replacing the reference (11) with one that is more in line with the paragraph.

RESPONSE: Done as suggested.

48. I suggest replacing bumblefoot for pododermatitis.

RESPONSE: Done as suggested.

128. What is RH set? Relative Humidity (RH)

RESPONSE: RH was defined as Relative Humidity.

165. A total of 396 colonies with different morphological... Was the selection of these colonies visual? Describe in the text.

RESPONSE: Done as suggested. A sentence was added specifying that colonies were selected visually by morphology and pigmentation.

187. 3.1. Bacterial identification on eggshell surface. What method was used to obtain these phylum-level results? The source and method is not clear in the text.

RESPONSE: We added that phylum distribution was derived from MALDI-TOF genus-level identifications and taxonomic mapping using curated databases.

188. I'm not sure if presenting the taxonomy at the phylum level is relevant to this work.

RESPONSE: Thank you for your comment. Presenting the taxonomy at the phylum level is relevant because it provides a stable and broadly comparable overview of the microbial community. Phylum-level patterns are standard in microbiome studies of eggshells and poultry environments, allowing readers to contextualize our results relative to the dominant microbial groups. In addition, this level offers greater taxonomic reliability, supporting the interpretation of broad community shifts before addressing finer classifications.

196. In Figure 2, the treatment Water presented 77.8% of Unidentified Genera. However, Figure 2 does not show this; it appears that unidentified genera represent approximately 52%.

RESPONSE: Dear Reviewer 4, thank you for this observation. This was a typo. Now we adjusted the number.

In Figure 2, why does the Dry treatment have 90%?

RESPONSE: This was an oversight, and Figure 2 has been corrected accordingly. We thank the reviewer for pointing this out.

Another issue is that the authors mentioning in line 195 that MALDI-TOF allowed the identification of 19 bacterial genera. However, Figure 2 shows 20 genera.

RESPONSE: Thank you for this important observation.

206. 3.1. Bacterial identification in yolk sac of newly hatched chicks. The topic number is not correct.

RESPONSE: Adjusted.

285-286. Mass spectrometry is not a method for bacteria isolation. I suggest rewrite this sentence.

RESPONSE: Rewritten to clarify that mass spectrometry was used only for bacterial identification.

In Figure 4, why does the Water treatment have 90%?

RESPONSE: This was an oversight, and Figure 4 has been corrected accordingly. We thank the reviewer for pointing this out.

It would be pertinent to attach the detailed protocol for identifying bacteria using MALDI-TOF, as this could be a valuable contribution from the authors of the study.

RESPONSE: We thank the reviewer for the suggestion. The bacterial identification by MALDI-TOF followed the same protocol previously described in Assis et al. (2019), from our research group, which provides a detailed methodological description. As this technique was not originally developed in the present study and was applied without modification, we believe that the citation of this reference is sufficient to ensure methodological clarity and reproducibility. Line 209.

The figures are not of good quality and need improvement. There are discrepancies between the text description and the figures. In Figure 2, I suggest representing the 10 most abundant genera (or pathogenic bacteria) to facilitate color selection. Plotting 19 different colors is difficult.

RESPONSE: We thank the reviewer for this comment. We acknowledge that discrepancies between the text and the figures were identified, and we would like to inform that other reviewers also pointed out these inconsistencies. All discrepancies between the text description and the figures have now been carefully corrected, as highlighted in yellow in the revised manuscript. Regarding Figure 2, although we recognize that representing a smaller number of genera could facilitate color distinction, we opted to maintain the visualization of all 19 identified genera. Studies focusing on the microbial characterization of the eggshell, and especially the yolk sac, remain very limited in the literature. Therefore, we believe that presenting the complete microbial profile provides valuable and comprehensive information. The figures were revised to improve their quality and readability, and we hope that the updated graphs now better meet the reviewer’s expectations.

It would be relevant to the work if the authors added an illustrative figure showing the different methods of egg sanitization. This way, readers can understand better.

RESPONSE: We thank the reviewer for the suggestion. The detailed methodology of the egg sanitization procedures is thoroughly described in Melo et al. (2019), which is cited in the manuscript. In the present study, we deliberately avoided reproducing this information in full to prevent redundancy with previously published work. This manuscript represents the third paper derived from the same doctoral thesis, and we aimed to maintain continuity while ensuring originality across the publications. Therefore, we believe that citing the original study where the sanitization methods were first detailed is sufficient for reader understanding, without the need to include an additional illustrative figure or a full methodological repetition.
